# Excess mortality in Russia and its regions compared to high income countries: An analysis of monthly series of 2020

**DOI:** 10.1016/j.ssmph.2021.101006

**Published:** 2021-12-17

**Authors:** Sergey Timonin, Ilya Klimkin, Vladimir M. Shkolnikov, Evgeny Andreev, Martin McKee, David A. Leon

**Affiliations:** a International Laboratory for Population and Health, National Research University Higher School of Economics, Myasnitskaya 20, 101000, Moscow, Russian Federation; b Laboratory of Demographic Data, Max Planck Institute for Demographic Research, Konrad-Zuse-Str. 1, 18057, Rostock, Germany; c Department of Health Services Research and Policy, London School of Hygiene & Tropical Medicine, Keppel St, London, WC1E 7HT, UK; d Department of Non-communicable Disease Epidemiology, London School of Hygiene & Tropical Medicine, Keppel St, London, WC1E 7HT, UK; e Department of Community Medicine, UiT Arctic University of Norway, Hansine Hansens veg 18, 9019, Tromsø, Norway

**Keywords:** CEE, Central and Eastern Europe

## Abstract

**Background:**

Russia has been portrayed in media as having one of the highest death tolls due to the COVID-19 pandemic in the world. However, the precise scale of excess mortality is still unclear. We provide the first estimates of excess mortality in Russia as a whole and its regions in 2020, placing this in an international context.

**Methods:**

We used monthly death rates for Russia and 83 regions plus the equivalent for 36 comparator countries. Expected mortality was derived in two ways using averages in the same months in preceding years and the same averages adjusted for secular trends. Excess death rates were estimated for the whole year and the last 3 quarters. We also estimated the relationships between excess mortality and reported COVID-19 cases and deaths across countries and Russian regions.

**Results:**

Estimating excess deaths rates based on the trend-adjusted average, Russia had the highest excess mortality of any of the 37 countries considered. Using the simple average, Russia had the third highest. Most of the excess deaths were recorded in the 4th quarter of 2020 and the level and trajectory of excess mortality in Russia and most of Eastern European countries differed from that in Western countries. While both the cumulative number of COVID-19 cases and deaths showed positive correlations with excess mortality across countries (r=0.65 and r=0.75, p<0.001), the association across the Russian regions was, surprisingly, negative for cases (r=-0.34, p<0.01) and deaths (r=-0.09, p=0.42). When we replaced reported deaths with final data from death certificates the correlation was positive (r=0.38, p<0.001).

**Conclusion:**

Russia has one of the largest absolute burden of excess mortality in 2020 but there is a counter-intuitive negative association between excess mortality and cumulative incidence at the regional level. Under-recording of COVID-19 cases seems to be a problem in some regions.

## Highlights

• Russia has experienced one of the highest excess mortality in 2020, particularly in the 4th quarter of the year.
• The patterns of excess mortality in Russia are most similar to those observed in most other CEE countries.
• Started in late spring in the two largest cities (Moscow and Saint Petersburg), Russian excess mortality spread to all regions of Russia by the end of 2020.
• The negative association between excess mortality and reported cases among Russian regions can be explained by under-recording of the latter.

## 1 Introduction

The past century has been remarkable for impressive progress in increasing longevity worldwide (Oeppen & Vaupel, 2002; Omran, 1971). There have been some setbacks (McMichael et al., 2004), including two world wars, the 1918 Spanish flu (Patterson & Pyle, 1991), the HIV/AIDS pandemic that brought devastation to sub-Saharan Africa in particular (Piot et al., 2001), and the mortality crisis in the countries of the former Soviet Union (FSU), beginning in the 1970s and continuing until the onset of rapid reductions in mortality in FSU countries in the late 1990s – early 2000s (Andreev et al., 2003; Grigoriev et al., 2014; Leon, 2011; Meslé, 2004; Shkolnikov et al., 2013; Timonin et al., 2016).

Alongside these overall positive trends, there have been repeated warnings by infectious disease experts of the risk posed by pandemics (Garrett, 1994; Jasilionis, 2018). Indeed it is now clear that the world was not prepared for the SARS-CoV-2 virus that emerged in China in late 2019, which caused over 1.8 million deaths directly related to COVID-19 by the end of 2020 (John Hopkins Coronavirus Resource Center, 2021).

The precise impact of the COVID-19 pandemic in Russia has been unclear, not least because of inconsistencies between official data sources. The Russian virus response center reported the cumulative number of COVID-19 deaths as 57,015 at the end of 2020 (Russian virus response center 2021). Yet the Federal State Statistical Service (Rosstat) reported more than 3 times as many deaths (163,325) based on preliminary death certificates where COVID-19 was listed as the underlying (104,826 deaths) or contributing (58,499 deaths) cause of death (Rosstat, 2021a). At the beginning of June 2021, Rosstat has reported the final number of deaths where COVID-19 is an underlying cause of death as 144,691 (+38% from the figure derived from preliminary death certificates and 2.5 times higher than reported by the Russian virus response center) (Rosstat, 2021c). The magnitude of this discrepancy generated many questions about what the true death toll was in Russia (Dyer, 2021).

Globally and in almost all countries the need to monitor the progress and impact of the pandemic has stimulated unprecedented efforts to collate and synthesise data in near real-time. However, from the beginning, there have been questions about the accuracy and international comparability of the data being reported. The quality of these data depended on the availability of tests, which were in very short supply early in the pandemic, on death registration, known to be patchy in many places, capacity of statistical offices to collate information on time, and government transparency, with some countries withholding data (Danilova, 2020).

The concept of “excess deaths” has been used extensively to enable comparisons of mortality directly or indirectly related to SARS-CoV-2 and is widely regarded as the gold standard (Beaney et al., 2020; Leon et al., 2020). It measures the additional number of deaths from all causes in any geographical area compared to what would be expected from the experience of mortality in previous years. It has at least three advantages. First, it is not sensitive to differences in cause-of- death coding practices. Second, it captures not just those deaths caused by infection with the virus, but also those that are either caused by or postponed by public health non-pharmaceutical interventions (such as lockdowns) aimed at reducing contact/infection rates in the population. Third, it is derived from vital registration data that is universally collected and collated in high income countries. Excess mortality has now been embraced as a key metric by many national statistical offices (Eurostat, 2021a; Office for National Statistics, 2021; Statistisches Bundesamt 2021), research organizations (Jdanov et al., 2021; Németh et al., 2021), international bodies (Morgan et al., 2020; EuroMOMO, 2021), analytic agencies (Karlinsky & Kobak, 2021; Our World in Data 2021) and leading media outlets (Financial Times, 2021).

Using harmonized mortality data disaggregated by age and sex from the Short-term Mortality Fluctuations (STMF) data series, Islam et al. have estimated that there were approximately one million excess deaths in 29 high income countries in 2020 (Islam et al., 2021). The highest excess death rates per 100,000 were in Lithuania, Poland, Spain, Hungary, Slovenia, Belgium, and Italy. Another study reported estimations of life expectancy losses in 2020 compared to 2019 for many of the same countries (Aburto et al., 2021). It found that life expectancy at birth declined in 25 of the 27 countries included, with the greatest falls in the USA, Bulgaria, Poland, Spain, and Lithuania.

Neither of these comparative studies includes Russia^1^. This is mainly because the Federal State Statistical Service of Russia (Rosstat) did not publish age-and-sex-specific mortality data by month or week for 2020 (Rosstat, 2021a). Karlinsky and Kobak remedy this situation, providing estimates of excess mortality for Russia and other 93 countries and territories with various quality of demographic data (Karlinsky & Kobak, 2021). In their latest release (April 4, 2021), Russia ranks fourth in terms of excess deaths per 100,000 behind Peru, Mexico and Bulgaria. However, comparisons are limited by differences in reporting lags. In this paper, we provide estimates of excess mortality for Russia as a whole and for its 83 regions, comparing it with 36 high income countries that provide high quality demographic data. To do this we apply two methods to estimate expected (baseline) mortality, the first based on averaging deaths rates for the preceding five years and the second adjusting this average for secular trends. First, we assess absolute and relative excess mortality by countries and regions of Russia for 2020. Second, we split our estimates into three quarters of the year to track the distribution of excess deaths across Russia and other countries. Third, we examine the association between excess death rates and cumulative number of reported COVID-19 cases and deaths per 100,000 for regions of Russia and other countries. To our knowledge, this is the first study looking at excess mortality in Russia at the regional level and placing it in an international perspective.

## 2 Methods

### 2.1 Data source

Our analysis focuses on Russia, its regions (N = 83) (we have excluded two, Crimea and the city of Sevastopol, because of the lack of data on earlier years) and 36^2^ high income countries. For Russia and 24 comparator countries, we obtained monthly death counts from national statistical offices. In the other 12 countries, we used weekly death counts obtained from the Short-Term Mortality Fluctuations (STMF) data series^3^ (Human Mortality Database, 2021) and transformed them to provide monthly numbers.^4^ Population exposures came from the Human Mortality Database (HMD) for countries and the Russian State Statistical Service (Rosstat, 2021a) for the regions of Russia.^5^

In the later part of our analysis, we used data on reported COVID-19 cases and deaths extracted from the Johns Hopkins Coronavirus Resource Center for countries including Russia (John Hopkins Coronavirus Resource Center, 2021) and from official reports for the regions of Russia (Russian Virus Response John Hopkins Coronavirus Resource Center, 2021). The definition of reported COVID-19 cases and deaths does differ among countries and has evolved over time so should be treated with caution (we discuss this issue in later in the paper). In Russia, the “reported cases” are those identified by a positive PCR test, registered by Rospotrebnadzor (the main hygienic and epidemiological center in Russia) and then reported by the regional governments to the Russian Virus Response Center. Deaths directly related to COVID-19 and reported daily by regional ministries of healthcare to the Russian Virus Response Center are described as “reported deaths” in Russia.

In the sensitivity analysis, we additionally used the following data that became available only recently for the regions of Russia: cumulative incidence rate (number of unique patients diagnosed with COVID-19 and treated in medical facilities in 2020)^6^ and death rates calculated from final civil registration data with COVID-19 as the underlying cause of death (RussianFertilityandMortalityDatabase, 2021).

### 2.2 Computing excess mortality measures

We used crude death rate (CDR) as a measure of mortality. This was calculated for each country/region, year and month by dividing the month-specific number of deaths adjusted for differences in the length of calendar months^7^ by the monthly population exposures:

(1) $${CDR}_{y,m,a}=\frac{D_{y,m,a}^{*}}{P_{y,a}/12}\;,$$

where *y* – year, *m* – month, *a* – country/region, D* – adjusted number of deaths, *P* – population exposure.

Two methods were used to estimate expected death rates (baseline mortality) in 2020. The first (denoted as “Method A″) corresponded to the most common approach, used widely by many organizations (England & Wales Office for National Statistics, Eurostat, OECD, etc.) and media teams (The Financial Times, The New York Times, The Economist, etc.) and is based on the averages in the same months over *N* preceding years. It is equivalent to a monthly fixed-effects model:

(2) $${CDR}_{m}^{A}={\hat{\alpha }}_{m}\;\text{,}$$

where the fixed effect estimates are derived from the model $CDR\left(t,m\right)=\alpha_{m}+\varepsilon_{t,m}\text{,}$ with *m* denoting months, and *t* denoting years: 2020-*N* ≤ *t* ≤ 2019.

The second approach (denoted as “Method B″) adjusted the first method for changes in annual crude death rates attributable to declining mortality and/or population aging. It is equivalent to the fixed effects model in Method A with an additional adjustment for the linear trend:

(3) $${CDR}_{m}^{B}={\hat{\alpha }}_{m}+\hat{\beta }∙y\text{,}$$

where estimates of the fixed effects and the slope are derived from the model $CDR\left(t,m\right)=\alpha_{m}+{\beta ∙t+\varepsilon }_{t,m}\text{.}$

We used data for 5 preceding years, i.e. 2015-2019, to estimate expected death rates for countries and regions of Russia. For Chile, Greece, and Germany we were limited to 4 years due to data availability.

As a sensitivity analysis we compared expected national death rates derived from Method B with the expected death rates predicted by Lee-Carter model (Lee & Carter, 1992), which is considered to be a “gold standard” to check the robustness of our estimates derived from Method B.

Absolute and relative measures of excess mortality for 2020 as a whole and three^8^ quarters of the year were calculated. The first is defined as a difference between observed and expected death rates per 100,000 population per year; the second is the ratio of absolute excess death rate to expected death rate. In our analysis, however, we mostly rely on absolute measures as they capture the scale of the burden of excess mortality. We explore country-specific patterns of excess mortality over the course of the year by depicting excess death rates in the second quarter (1st wave of the pandemic) vs the fourth quarter (2nd wave).

We used bootstrapping techniques to derive 95% confidence limits for annual expected and excess death rates.

### 2.3 Estimating the association between excess mortality and COVID-19 cases and deaths

In order to explore the coherence and validity of the data on COVID-19 in Russia we first examined the associations between excess mortality and officially reported COVID-19 cases and deaths across regions of Russia and across countries. To do this we regressed (OLS models) the excess death rates on cumulative number of cases and on COVID-19 deaths (per 100,000) separately for regions and countries. As a sensitivity analysis, we then regressed the excess death rates on cumulative incidence rates (number of COVID patients attending medical facilities) and on death rates where COVID-19 was the underlying cause of death in the final medical death certificate.

Geographical maps were built in ArcGIS, Esri (10.4). We constructed choropleth maps and cluster maps based on Anselin Moral I statistics (Anselin, 1995) for three quarters of 2020 and for the whole year. Statistical analysis was performed in R Studio (4.0.3).

## 3 Results

### 3.1 Ranking of countries by excess death rates

**Fig. 1:**
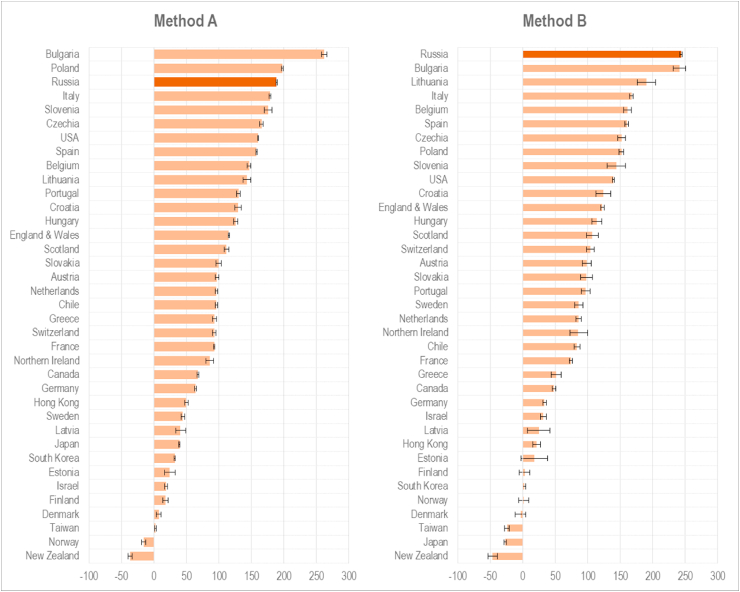
Excess death rates in 2020 estimated by two methods, per 100,000. Note: Method A – averages in the same months in preceding years; Method B – averages adjusted for secular trends.

Excess death rates for Russia and other countries in 2020 are presented in Fig. 1 and Table A1 in Appendix. The absolute excess death rates in Russia, estimated using methods A and B, are 189 (95%CI: 188, 190) and 244 (95%CI: 242, 246) per 100,000, respectively. Depending on the method, Russia ranks as the third after Bulgaria and Poland (method A) or first (method B) among 37 comparator countries ranked by overall excess deaths per 100,000 in 2020 (Fig. 1). Other countries with a high burden of excess mortality in 2020 are Central and Eastern European states (Bulgaria, Poland, Lithuania, Slovenia, and Czechia) followed by several Southern and Western European countries (Italy, Spain, and Belgium) and the United States. Countries of Eastern Asia and Pacific region (Japan, Taiwan, South Korea, and New Zealand) as well as Scandinavian countries (Norway, Finland and Denmark) did not experience elevated mortality in 2020. Estonia and Latvia are also among countries with very low excess mortality in 2020 (Fig. 1 and Table A1 in Appendix).

According to method B, for 2020 as a whole Russia experienced the highest relative excess mortality of any of the countries we analysed (20.1% (95%CI: 19.9, 20.3)). The relative position of other countries roughly follows the ranking of countries by absolute excess death rates (Table A1 in Appendix).

Adjustment for secular trend (method B) makes especially important differences from the average-based estimates (method A) for populations that had experienced a steep decline in crude death rates (Russia and some other CEE countries) or increase in crude death rates due to rapid population aging (Japan, for instance) prior to 2020. Given that method B gives more internationally comparable results the remainder of our analyses are based on estimates obtained using this method only.

### 3.2 Excess mortality by the quarters of 2020

The largest increase in all-cause mortality in Russia was recorded in the fourth quarter of the year, with 636 (95%CI: 634, 639) excess deaths per 100,000. This compares with a smaller but still large relative excess mortality in Russia in the second and the third quarters of the year, at 82 (95%CI: 79, 84) and 236 (95%CI: 234, 238) per 100,000, respectively.

**Fig. 2:**
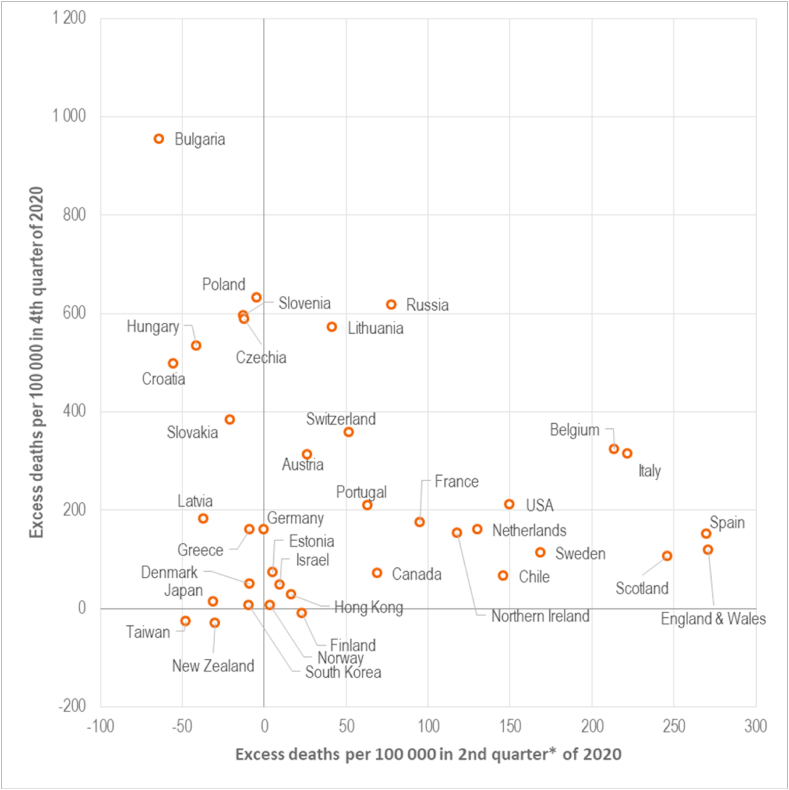
Association between excess death rates in the second and in the fourth quarters of 2020. Notes: * second quarter includes 4 months – March–June of 2020.

Fig. 2 shows clustering of the countries depending on when their populations experienced the largest excess death hazard in 2020. The majority of CEE countries are located in the top left-hand section of the plot, with some of the highest excess death rates in the 4th quarter of the year, but negative or only marginally increased excess mortality in 2nd quarter. This pattern is different to that observed in most of the Western countries. Parts of the UK, Spain, Belgium, Italy, the Netherlands, the USA, and Sweden present the clearest contrast to CEE countries with most of their excess mortality concentrated within the 2nd quarter.

### 3.3 Geographical patterns of excess mortality in Russia

**Fig. 3:**
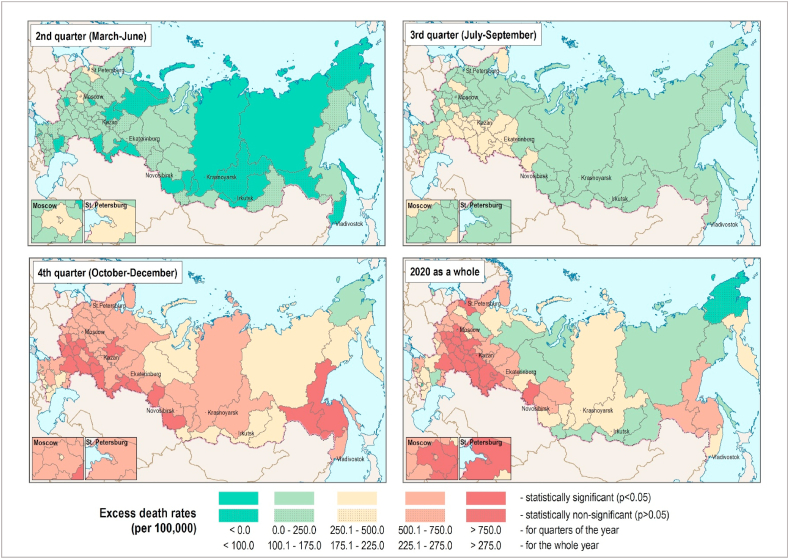
Excess deaths rates across region of Russia, by quarters of 2020 and for the whole year. Note: data for Crimea and city of Sevastopol are not available.

**Fig. A1:**
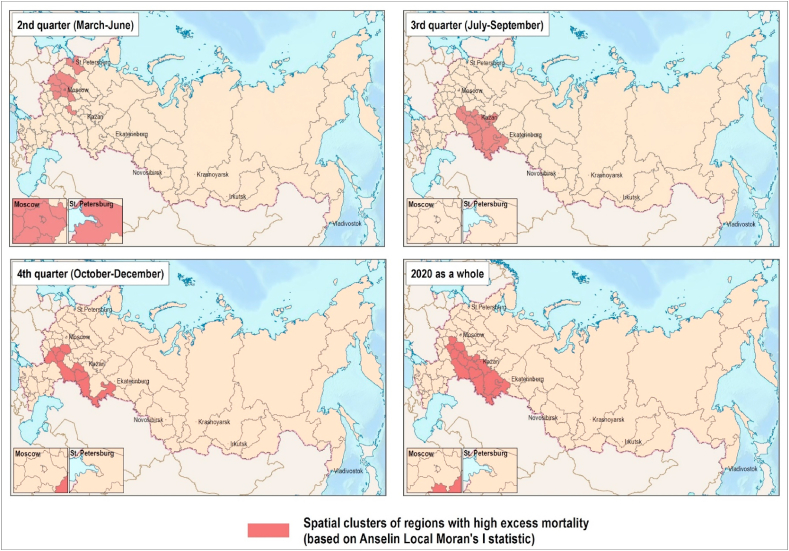
Spatial clusters of high excess deaths rates in Russia, by quarters of 2020 and for the whole yearNote data for Crimea and city of Sevastopol are not available.

Spatial and temporal patterns of excess death rates across regions of Russia are presented in Fig. 3 and Figure A1 in Appendix. The mortality increase started from the two metropolitan areas (the city of Moscow and Moscow region, and Saint Petersburg and Leningrad region). Each had very similar excess death rates in the second quarter of 2020 (Fig. 3, left upper corner). In the third quarter, the list of regions experiencing elevated mortality has expanded, forming a large spatial cluster in the south of the Volga and Urals regions (Fig. 3 and Figure A1, right upper corner). Interestingly, Moscow and Saint Petersburg regions have managed to contain and even reduce excess mortality in the third quarter. In the fourth quarter, the COVID-19 pandemic spread to all regions of Russia (Fig. 3, left lower corner). The highest absolute excess death rates in 2020 were observed in a big cluster of territories located southeast of Moscow (Figure A1 in Appendix), especially in some regions of the Volga, South of the Urals and Center of European Russia (see Table A2 in Appendix for more details). The highest relative excess death rates, however, were observed in the republics of North Caucasus (Chechnya, Dagestan and Ingushetia) having had the lowest baseline mortality levels.

### 3.4 Correlation with COVID cases and deaths

**Fig. 4:**
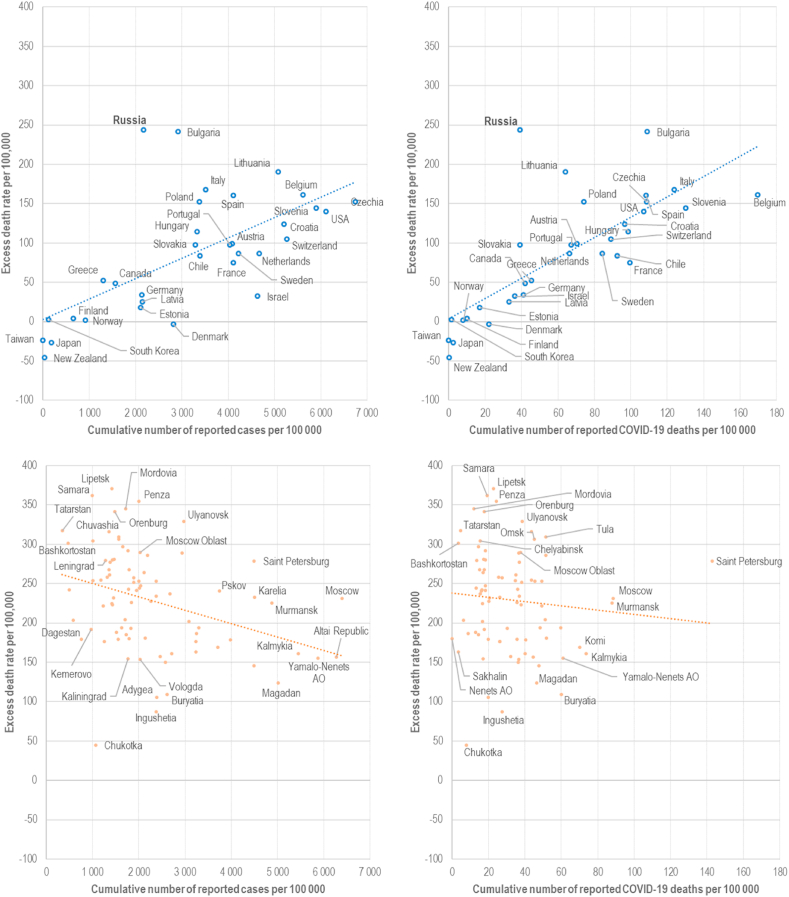
Association between excess death rates and cumulative number of a) reported COVID cases (left panel) and b) reported COVID deaths (right panel) in 2020, by countries (upper panel) and regions of Russia (lower panel). Note: linear regression lines for countries and for regions are depicted on the graphs.

Fig. 4 (left panel) shows intriguing associations between cumulative numbers of reported COVID-19 cases per 100,000 and excess death rates across countries and across regions of Russia. With countries as the unit of analysis, there is an expected positive correlation between these indicators, with Pearson's correlation coefficient equal to 0.65 (p < 0.001). Russia and Bulgaria, however, are outliers, with excess deaths rates much higher than could be expected given the numbers of disease cases.

The correlation across regions of Russia is, on the contrary, negative (r = -0.34, p < 0.001) which seems counter-intuitive.^9^ We also undertake separate analyses with data from the last three quarters of 2020. This yields a positive association in the second quarter (r = 0.39, p < 0.001), and negative associations in both third (r = -0.24, p < 0.05) and fourth (r = -0.30, p < 0.01) quarters. The correlation between reported COVID-19 deaths (per 100,000) and excess death rates is also positive for countries (r = 0.75, p < 0.001) but again negative (r = -0.09, p = 0.42) for the regions of Russia (Fig. 4, right panel).

Figure A3 (left panel) shows the results of the sensitivity analysis where we additionally regress excess death rates on cumulative incidence rate, i.e. number of patients (r = -0.27, p = 0.014). Both the data on reported cases and on unique patients diagnosed with COVID-19 in outpatient or inpatient settings show negative correlation with excess mortality at the regional level in Russia. When we use the final data on COVID deaths from the medical death certificates instead of daily reported deaths, the association with excess mortality becomes positive, with Pearson's correlation coefficient equal to 0.38 (p < 0.001).

## 4 Discussion

### 4.1 Summary of main findings

Compared to a group of high income countries with reliable demographic statistics, Russia experienced one of the highest rates of excess mortality in 2020. After the first peak in late spring/beginning of summer, concentrated in the two largest cities (Moscow and Saint Petersburg), Russian excess death rates dropped as strict quarantine measures were introduced and the health system was mobilized at national and regional levels. September marked the beginning of an upsurge in excess mortality in Russia to levels that persisted until the end of the year. During the second wave, excess mortality had spread to all regions of Russia, and especially those to the southeast of Moscow. Regions in the Volga Federal District and the south of the Ural, as well as some regions in the center of European Russia and Siberian Federal Districts were most severely affected during 2020. The city of Moscow, which was an epicenter of the COVID-19 pandemic during the first wave, did manage to resist the pandemic in the second half of the year, with only moderate excess mortality (30% lower than the Russian average).

The patterns of excess mortality in Russia and most of Central and Eastern European countries are quite distinct to those observed in Western counties. CEE countries experienced negative or slightly elevated excess mortality during the first wave of the pandemic but some of the highest excess death rates during the second wave. This was quite different from what was seen in many Western countries, that were badly hit in spring but managed to avoid a large increase in autumn/winter of 2020. There are likely to be many factors involved. The initial spread from China was to northern Italy, arriving in the middle of the ski season when this region attracted tourists, especially from Western Europe, who took the infection back to their home countries. There was also early spread to Spain associated with a football match (Sassano et al., 2020), with cases then spreading to other Northern European countries. However, there was less spread to Central and Eastern Europe, likely reflecting in part a lower volume of travel from Italy and Spain but also the rapid imposition of stringent restrictions, typically before many cases had arisen, including imposition of, and widespread adherence to mask wearing (Walker & Smith, 2020). This is likely to have had an additional benefit in reducing spread of other seasonal respiratory viruses.

Negative correlation between cumulative numbers of cases and excess deaths across regions of Russia contrasted with positive association across countries (and within some other countries such as the USA and France) is an unexpected and interesting finding of this study. On the one hand, high excess mortality in regions with low reported numbers of cases might be explained by their inadequate capacity to test and thus identify new cases of diseases. On the other hand, reporting of new cases could also vary considerably amongst the regions. The persistence of a negative correlation between excess death rates and incidence rates, i.e. number of unique patients diagnosed with COVID and treated in medical facilities, across regions of Russia further strengthens the suggestion from our findings that, on average, regions with the lowest levels of reported cases had the lowest number of treated patients and highest excess mortality.

The observed negative correlation between excess death rates and reported COVID-19 mortality across the regions of Russia is unexpected and could be only explained by problems with post mortem diagnostic, coding practices, and reporting of COVID-19 deaths to the Russian virus response center. Final cause-of-death statistics of 2020 showed that correlation becomes positive (but still not very high).

### 4.2 Are there alternative approaches to measure COVID-related human losses?

One might also assess the impact of the COVID-19 pandemic by means of life expectancy losses or other related measures. Preliminary estimates of life expectancy at birth for Russia and its regions have been recently published by the Rosstat (Rosstat, 2021b) but they have several shortcomings. First, the age- and sex-specific deaths rates used to construct life tables were not available at the time of writing this paper. Secondly, life expectancy measure cannot be used to assess human losses by the quarters of the year. Nevertheless, a life expectancy decrease of 1.8 year in Russia (compared with 2019) is higher than in any of the EU Member States (Eurostat, 2021b) but may be slightly lower than that in the USA (Aburto et al., 2021). The largest decrease in life expectancy in EU has been recorded in Bulgaria (-1.7 years) which corresponds with our ranking of countries by the overall excess mortality.

### 4.3 Which approach should be used to estimate baseline mortality?

The magnitude of excess death rates and thus their comparative rankings are dependent on the methods used to estimate baseline mortality. In this study, we apply two commonly used approaches to the definition of excess mortality. Method A, based on simple averaging over previous years is the most popular approach allowing direct comparisons with earlier reports, though the number of preceding years used for averaging might be different. This method works well if the death rate is fluctuating around the same level over the reference period. However, this method is suboptimal when death rates are steeply decreasing (as in Russia or some other countries in Eastern Europe) or increasing (as in the aging populations of Japan). In the former examples, excess mortality will be understated while in the latter it will be overstated. Method B, with its additional adjustment for linear trend, allows us to better capture the recent changes in mortality and aging and thus achieve a better fit of the observed dynamics in many countries.

### 4.4 Comparison with previous results

High excess mortality attributable to the COVID-19 pandemic in Russia has previously been highlighted in the media (Financial Times, 2021) and can be found in the World Mortality Dataset (Karlinsky & Kobak, 2021). However, it has not been systematically examined or reported in peer-reviewed journals. These other reports note that Russia has one of the world's highest excess death rates (behind some countries in Latin America) and Bulgaria among the CEE. These results generally correspond with our estimations. Nevertheless, none of the studies examines excess mortality in Russia and its regions in 2020 from an international perspective and in association with reported COVID cases and deaths.

### 4.5 What explains the situation in Russia?

Russia, like many other countries, has implemented various non-pharmaceutical interventions (NPIs) designed to reduce transmission of the SARS-CoV-2 virus. However, they were relatively weak compared to many other countries. National restrictions began in February 2020, with bans on flights from several countries such as China, the Republic of Korea, and Iran. A national lockdown, i.e. closure of offices and factories accompanied by severe travel restrictions was then implemented during spring 2020 (from March 30, when the 7 day average of new cases was 200, until May 8, 2020, when it had risen to 10,490, almost but not at the peak of the first wave). Regional authorities had already been adopting an increasing role in implementing further restrictions (Executive Order, 2020). Starting with Moscow, all other regions have introduced the so-called “high alert” regime, which allows them to impose restrictions according to the current epidemiological situation in each particular region (Moscow did so on March 5th, 2020). In fact, most regional authorities adopted those NPIs first implemented in the city of Moscow.

Large scale regional interventions, such as recommendations or obligations to work from and stay at home (particularly for those 65+), special travel passes, cancellations of public events, closure of schools, museums, restaurants and fitness centres were introduced in late March/beginning of April 2020. Most of these measures were then lifted gradually during the summer as the number of new cases and deaths declined. Though the measures adopted were more or less the same across the regions of Russia, the duration and strictness of their implementation differed.

The second wave however did not prompt the Russian authorities to re-introduce the same NPIs in autumn. Regional governments have only implemented some policies aimed at curbing human contacts from mid-autumn 2020 until mid-winter 2021. The majority of them are distance learning for students at schools and universities, very mild restrictions on the operation of restaurants at night, recommendations for elderly people to stay at home, and for employers to transfer some employees to remote work (Plaksin et al., 2021).

**Fig. A2:**
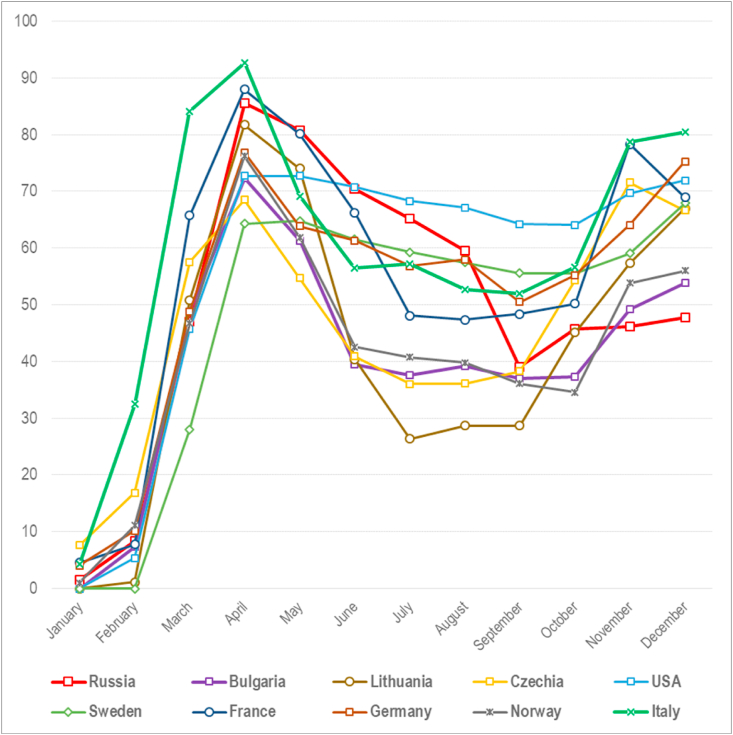
Stringency index in countries with different levels of excess mortality in 2020 Source: Blavatnik School of BlavatnikSchool of Government, 2021.

**Fig. A3:**
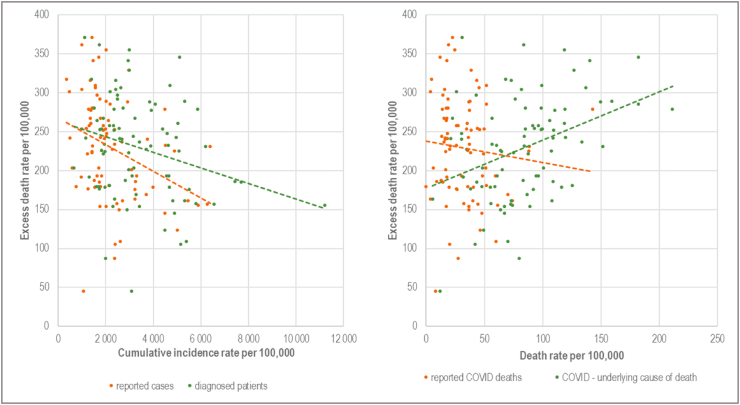
Association between excess death rates and a) cumulative incidence rates (left panel) and b) death rates (right panel) in 2020, by regions of Russia (lower panel). Note: linear regression lines for countries and for regions are depicted on the graphs.

The timing of the NPIs in Russia and comparator countries over the year can be seen in the Blavatnik Government Response Stringency Index (SI) - an objective measure of the overall intensity of restrictions ranging from 0 (no restrictions) to 100 (strictest restrictions) (BlavatnikSchool of Government, 2021). Figure A2 in Appendix confirms that the strongest restrictions in Russia (SI = 81 to 85) were applied in April–May 2020. In the last quarter of the year, when new reported daily cases and deaths were increasing, the stringency index was around 47. At the same time, in most European countries, SI has substantially risen September–December 2020 as depicted in Figure A2 in Appendix.

The Russian Healthcare system has been mobilized. This is led by regional authorities, working within guidance (Ministry of Health of the Russian Federation, 2021a) and with financial support from the federal government. The Ministry of Finance reports that federal expenditure on healthcare increased 1.9 times in 2020 compared to 2019, and amounted to 5.8% of all federal expenditure up from 3.9% in 2019 (Ministry of Finance of the Russian Federation, 2021). The additional sums were mainly transfers to regions to provide medical care for patients with COVID- 19 and incentive payments to medical professionals working with these patients.

The Ministry of Health reports that 2450 hospitals provided medical care to patients with COVID-19, including 40 newly constructed temporary hospitals. More than 40,000 beds in infectious disease hospitals and 235,000 beds in repurposed medical facilities were mobilized to treat patients. Doctors have been diverted to treat patients with COVID-19 following short training programs regardless of their primary specialization (Ministry of Health of the Russian Federation, 2021b). However, we lack detailed information on what has happened in individual regions.

In August 2020, Russia registered the world's first vaccine based on a human adenoviral vector-based platform Sputnik V. After the third phase of clinical trials, vaccine efficacy was measured at 91.6% (95% CI: 85.6, 95.2) (Logunov et al., 2021). The mass vaccination in Russia started in January 2021. On April 23, Deputy Prime Minister Tatyana Golikova, at a meeting with Vladimir Putin, said that more than 11.1 million Russians received at least one dose of the coronavirus vaccine, both doses received 6.8 million (4.7% of the total population).

### 4.6 Limitations of the study

This study has limitations. The most important is that we had to use aggregate crude death rates that depend on age-specific death rates and population age structures. At the time of writing, mortality data disaggregated by age and sex were not available for Russia and some other comparator countries. It would be interesting to carry out an analysis of mortality patterns by age and sex as soon as the necessary data are available.

The second limitation concerns the linear adjustments used in method B. Due to the fact that changes of crude death rates are the interplay of changing mortality and population ageing, it is rather challenging to model them with a linear trend. To elaborate on this issue, we have conducted sensitivity analysis using the “gold standard” Lee-Carter method to predict crude death rates for all countries including Russia. Excess deaths rates derived from method B and its modification with Lee-Carter prediction show very similar results with Spearman's r = 0.97 (95% CI: 0.96, 0.98) and Kendall's τ = 0.87 (95% CI: 0.84, 0.90).

Finally, we used only countries with reliable demographic statistics (those included in the Human Mortality Database) as comparators in our study. We do not have reliable estimates of excess mortality in 2020 as the Word Mortality Dataset suggests (Karlinsky & Kobak, 2021), several countries in Latin America and elsewhere might have had excess death rates higher than those observed in Russia in 2020.

## Ethical statement

The study is based on publicly available data, and as such it did not require ethical approval.

## Declaration of competing interest

The authors declare no competing interests.

## Funding sources

The study was partly funded by the Ministry of Science and Higher Education of the Russian Federation (grant ID: 075-15-2020-928).

## CRediT authors contribution statement

**ST**: Writing-Original draft preparation, Reviewing and Editing, Visualization. **IK**: Data Curation, Formal analysis. **VMS:** Conceptualization, Writing- Reviewing and Editing. **EA**: Writing-Reviewing and Editing. **MM**: Writing-Reviewing and Editing. **DAL**: Writing-Reviewing and Editing.

## Figures and Tables

**Table A1:** Observed and excess deaths in 2020 in Russian and 36 comparator high income countries

#	Country	Observed deaths per 100,000	Excess death rates per 100,000 (95% confidence interval)		Excess death rates to expected death rates in % (95% confidence interval)		Excess deaths in thousands (95% confidence interval)	
			Method B*	Method A	Method B	Method A	Method B	Method A
1	Russia	1454	244 (242, 246)	189 (188, 190)	20.1 (19.9, 20.3)	14.9 (14.9, 15)	350.1 (347.4, 352.9)	271.6 (270.4, 272.7)
2	Bulgaria	1801	241 (232, 250)	262 (258, 267)	15.4 (14.8, 16.1)	17.1 (16.8, 17.4)	16.7 (16.1, 17.4)	18.2 (17.9, 18.5)
3	Lithuania	1557	190 (176, 205)	143 (137, 149)	13.9 (12.8, 15.1)	10.1 (9.7, 10.6)	5.3 (4.9, 5.7)	4 (3.8, 4.2)
4	Italy	1230	168 (165, 170)	179 (178, 180)	15.8 (15.5, 16.1)	17 (16.9, 17.1)	100.3 (98.6, 101.8)	107 (106.3, 107.7)
5	Belgium	1108	161 (156, 167)	147 (144, 149)	17 (16.3, 17.8)	15.3 (14.9, 15.6)	18.5 (17.9, 19.2)	16.9 (16.6, 17.1)
6	Spain	1055	160 (157, 163)	158 (157, 159)	17.9 (17.5, 18.3)	17.6 (17.5, 17.8)	75.1 (73.8, 76.4)	74.1 (73.5, 74.7)
7	Czechia	1214	152 (146, 159)	166 (163, 168)	14.4 (13.7, 15)	15.8 (15.5, 16.1)	16.2 (15.6, 16.9)	17.6 (17.4, 17.9)
8	Poland	1243	152 (149, 156)	197 (196, 199)	13.9 (13.6, 14.3)	18.9 (18.7, 19)	58.4 (57, 59.7)	75.7 (75.2, 76.2)
9	Slovenia	1155	144 (130, 158)	176 (170, 182)	14.3 (12.7, 15.8)	18 (17.3, 18.7)	3 (2.7, 3.3)	3.6 (3.5, 3.8)
10	USA	1021	140 (138, 141)	161 (160, 161)	15.8 (15.7, 16)	18.7 (18.6, 18.7)	458.3 (454.8, 462.1)	527.4 (525.8, 528.9)
11	Croatia	1406	124 (112, 135)	130 (124, 135)	9.7 (8.7, 10.7)	10.2 (9.7, 10.6)	5 (4.6, 5.5)	5.3 (5, 5.5)
12	England & Wales	1022	123 (120, 125)	115 (114, 116)	13.6 (13.3, 14)	12.7 (12.6, 12.8)	72.9 (71.4, 74.5)	68.6 (67.9, 69.2)
13	Hungary	1457	114 (106, 122)	126 (123, 129)	8.5 (7.9, 9.1)	9.4 (9.2, 9.7)	11 (10.3, 11.8)	12.2 (11.9, 12.5)
14	Scotland	1178	107 (99, 116)	112 (108, 116)	10 (9.1, 10.9)	10.5 (10.1, 10.9)	5.8 (5.4, 6.3)	6.1 (5.9, 6.3)
15	Switzerland	886	104 (98, 110)	93 (91, 96)	13.3 (12.5, 14.2)	11.8 (11.4, 12.1)	8.9 (8.4, 9.4)	8 (7.8, 8.2)
16	Austria	1042	99 (92, 106)	97 (94, 100)	10.5 (9.7, 11.3)	10.3 (9.9, 10.6)	8.7 (8.1, 9.3)	8.6 (8.3, 8.8)
17	Slovakia	1084	98 (89, 107)	99 (95, 103)	9.9 (8.9, 10.9)	10.1 (9.6, 10.5)	5.3 (4.9, 5.8)	5.4 (5.2, 5.6)
18	Portugal	1204	97 (90, 104)	130 (127, 133)	8.8 (8.1, 9.4)	12.1 (11.8, 12.4)	9.9 (9.2, 10.6)	13.3 (13, 13.6)
19	Sweden	950	86 (80, 93)	45 (42, 47)	10 (9.3, 10.8)	4.9 (4.6, 5.2)	8.9 (8.3, 9.6)	4.6 (4.3, 4.9)
20	Netherlands	974	86 (82, 90)	96 (95, 99)	9.7 (9.1, 10.2)	11 (10.7, 11.3)	14.9 (14.1, 15.6)	16.7 (16.4, 17)
21	Northern Ireland	927	85 (72, 100)	86 (80, 92)	10.1 (8.5, 12.1)	10.3 (9.5, 11)	1.6 (1.4, 1.9)	1.6 (1.5, 1.7)
22	Chile	702	84 (79, 88)	96 (95, 98)	13.5 (12.7, 14.4)	15.9 (15.6, 16.3)	15 (14.2, 15.8)	17.3 (17, 17.6)
23	France	1006	74 (72, 77)	93 (92, 94)	8 (7.7, 8.3)	10.2 (10.1, 10.3)	48.4 (46.9, 49.9)	60.5 (59.8, 61.1)
24	Greece	1232	52 (44, 60)	94 (90, 97)	4.4 (3.7, 5.1)	8.2 (7.9, 8.5)	5.5 (4.6, 6.4)	10 (9.6, 10.3)
25	Canada	821	48 (45, 51)	68 (67, 69)	6.2 (5.8, 6.6)	9.1 (8.9, 9.2)	18.1 (17, 19.1)	25.5 (25, 26)
26	Germany	1196	34 (31, 36)	64 (63, 66)	2.9 (2.6, 3.1)	5.7 (5.6, 5.8)	27.6 (25.3, 29.9)	53 (52.1, 54)
27	Israel	536	32 (27, 37)	19 (17, 21)	6.4 (5.4, 7.4)	3.6 (3.2, 4.1)	2.9 (2.5, 3.4)	1.7 (1.5, 1.9)
28	Latvia	1505	25 (8, 42)	41 (34, 49)	1.7 (0.5, 2.9)	2.8 (2.3, 3.4)	0.5 (0.1, 0.8)	0.8 (0.6, 0.9)
29	Hong Kong	697	22 (16, 28)	50 (48, 53)	3.2 (2.3, 4.2)	7.8 (7.3, 8.2)	1.6 (1.2, 2.1)	3.7 (3.5, 3.9)
30	Estonia	1197	18 (-2, 38)	25 (17, 33)	1.5 (-0.2, 3.3)	2.1 (1.4, 2.8)	0.2 (0, 0.5)	0.3 (0.2, 0.4)
31	Finland	993	4 (-5, 11)	18 (14, 22)	0.4 (-0.5, 1.2)	1.8 (1.5, 2.2)	0.2 (-0.3, 0.6)	1 (0.8, 1.2)
32	South Korea	593	3 (0, 5)	32 (31, 33)	0.4 (0.1, 0.8)	5.8 (5.6, 5.9)	1.3 (0.2, 2.4)	16.6 (16.1, 17.1)
33	Norway	758	1 (-6, 9)	-15 (-19, -12)	0.2 (-0.8, 1.2)	-2 (-2.4, -1.6)	0.1 (-0.3, 0.5)	-0.8 (-1, -0.6)
34	Denmark	938	-3 (-12, 5)	8 (4, 11)	-0.4 (-1.2, 0.6)	0.8 (0.5, 1.2)	-0.2 (-0.7, 0.3)	0.5 (0.3, 0.7)
35	Taiwan	730	-24 (-28, -21)	2 (1, 4)	-3.2 (-3.6, -2.8)	0.3 (0.1, 0.5)	-5.7 (-6.5, -4.9)	0.5 (0.2, 0.9)
36	Japan	1112	-27 (-29, -25)	40 (39, 40)	-2.4 (-2.5, -2.2)	3.7 (3.6, 3.8)	-33.1 (-35.5, -30.8)	48.8 (47.8, 49.8)
37	New Zealand	651	-46 (-54, -39)	-36 (-40, -33)	-6.6 (-7.6, -5.6)	-5.3 (-5.8, -4.9)	-2.3 (-2.7, -1.9)	-1.8 (-2, -1.7)

Notes: Method A – averages in the same months in preceding years; Method B – averages adjusted for secular trends.

* Countries are listed in descending order by excess death rates (method B).

**Table A2:** Observed and excess death in 2020 in 83 regions of Russia

#	Region	Observed deaths per 100,000	Excess death rates per 100,000 (95% confidence interval)		Excess death rates to expected death rates in % (95% confidence interval)		Excess deaths in thousands (95% confidence interval)	
			Method B*	Method A	Method B	Method A	Method B	Method A
1	Lipetsk	1767	371 (349, 392)	289 (278, 298)	26.6 (24.6, 28.5)	19.5 (18.7, 20.3)	4.2 (4, 4.5)	3.3 (3.2, 3.4)
2	Samara	1662	362 (347, 377)	291 (286, 297)	27.8 (26.4, 29.3)	21.3 (20.8, 21.7)	11.5 (11, 11.9)	9.2 (9.1, 9.4)
3	Penza	1742	355 (334, 376)	302 (293, 310)	25.6 (23.7, 27.6)	21 (20.2, 21.7)	4.6 (4.3, 4.9)	3.9 (3.8, 4)
4	Mordovia	1638	345 (317, 373)	272 (261, 284)	26.7 (24, 29.5)	19.9 (18.9, 21)	2.7 (2.5, 2.9)	2.1 (2.1, 2.2)
5	Orenburg	1619	341 (324, 357)	276 (269, 283)	26.7 (25, 28.3)	20.5 (19.9, 21.2)	6.7 (6.3, 7)	5.4 (5.2, 5.5)
6	Ulyanovsk	1683	329 (309, 351)	249 (240, 259)	24.3 (22.4, 26.3)	17.4 (16.6, 18.2)	4 (3.8, 4.3)	3.1 (2.9, 3.2)
7	Tatarstan	1398	318 (307, 329)	250 (245, 255)	29.4 (28.2, 30.7)	21.8 (21.3, 22.3)	12.4 (12, 12.8)	9.8 (9.6, 9.9)
8	Chuvashia	1538	316 (296, 336)	258 (249, 267)	25.9 (23.9, 27.9)	20.2 (19.3, 21)	3.8 (3.6, 4.1)	3.1 (3, 3.2)
9	Tula	1864	309 (287, 330)	211 (202, 221)	19.9 (18.2, 21.5)	12.8 (12.2, 13.4)	4.5 (4.2, 4.8)	3.1 (2.9, 3.2)
10	Omsk	1550	306 (289, 322)	251 (244, 258)	24.6 (23, 26.2)	19.3 (18.6, 19.9)	5.9 (5.6, 6.2)	4.8 (4.7, 5)
11	Chelyabinsk	1581	305 (292, 316)	245 (239, 250)	23.9 (22.6, 25)	18.3 (17.8, 18.8)	10.5 (10.1, 10.9)	8.5 (8.3, 8.6)
12	Bashkortostan	1483	301 (290, 313)	221 (216, 226)	25.5 (24.3, 26.8)	17.5 (17.1, 17.9)	12.1 (11.7, 12.6)	8.9 (8.7, 9.1)
13	Ryazan	1793	297 (274, 321)	241 (231, 251)	19.9 (18, 21.8)	15.5 (14.8, 16.3)	3.3 (3, 3.5)	2.7 (2.6, 2.8)
14	Tambov	1772	292 (268, 315)	219 (209, 230)	19.7 (17.8, 21.6)	14.1 (13.4, 14.9)	2.9 (2.7, 3.2)	2.2 (2.1, 2.3)
15	Moscow Oblast	1456	289 (281, 297)	208 (204, 211)	24.8 (23.9, 25.7)	16.6 (16.3, 17)	22.2 (21.6, 22.8)	15.9 (15.7, 16.2)
16	Oryol	1803	289 (259, 317)	208 (196, 221)	19.1 (16.8, 21.3)	13.1 (12.2, 14)	2.1 (1.9, 2.3)	1.5 (1.4, 1.6)
17	Nizhny Novgorod	1724	286 (271, 300)	218 (212, 224)	19.9 (18.7, 21.1)	14.5 (14, 15)	9.1 (8.6, 9.6)	7 (6.8, 7.2)
18	Saratov	1638	281 (264, 296)	249 (242, 255)	20.7 (19.2, 22)	17.9 (17.3, 18.5)	6.8 (6.4, 7.1)	6 (5.8, 6.2)
19	Volgograd	1572	280 (265, 294)	231 (225, 238)	21.7 (20.3, 23)	17.3 (16.7, 17.8)	7 (6.6, 7.3)	5.7 (5.6, 5.9)
20	Leningrad	1494	279 (262, 296)	159 (151, 167)	23 (21.3, 24.7)	11.9 (11.3, 12.6)	5.2 (4.9, 5.5)	3 (2.8, 3.1)
21	Saint Petersburg	1350	279 (270, 288)	206 (202, 210)	26.1 (25, 27.1)	18 (17.6, 18.5)	15 (14.5, 15.5)	11.1 (10.9, 11.3)
22	Vladimir	1811	278 (255, 300)	206 (197, 215)	18.1 (16.4, 19.8)	12.9 (12.2, 13.5)	3.7 (3.4, 4.1)	2.8 (2.7, 2.9)
23	Mari El	1431	268 (241, 295)	149 (137, 160)	23 (20.3, 25.9)	11.6 (10.6, 12.6)	1.8 (1.6, 2)	1 (0.9, 1.1)
24	Yaroslavl	1709	267 (246, 290)	187 (177, 196)	18.6 (16.8, 20.4)	12.3 (11.6, 13)	3.3 (3.1, 3.6)	2.3 (2.2, 2.4)
25	Kaluga	1719	264 (239, 290)	232 (221, 242)	18.2 (16.2, 20.3)	15.6 (14.7, 16.4)	2.6 (2.4, 2.9)	2.3 (2.2, 2.4)
26	Sverdlovsk	1563	261 (250, 273)	195 (190, 200)	20 (19, 21.1)	14.3 (13.9, 14.7)	11.2 (10.7, 11.7)	8.4 (8.2, 8.6)
27	Udmurtia	1405	258 (241, 275)	179 (171, 186)	22.5 (20.7, 24.4)	14.6 (13.9, 15.3)	3.9 (3.6, 4.1)	2.7 (2.6, 2.8)
28	Kursk	1728	258 (233, 281)	161 (151, 171)	17.5 (15.6, 19.4)	10.3 (9.6, 11)	2.8 (2.6, 3.1)	1.8 (1.7, 1.9)
29	Perm	1546	255 (240, 270)	187 (180, 193)	19.7 (18.3, 21.2)	13.7 (13.2, 14.3)	6.6 (6.2, 7)	4.8 (4.7, 5)
30	Novosibirsk	1524	254 (240, 267)	230 (224, 236)	20 (18.7, 21.3)	17.8 (17.2, 18.4)	7.1 (6.7, 7.5)	6.4 (6.3, 6.6)
31	Voronezh	1647	253 (238, 268)	168 (161, 174)	18.2 (16.9, 19.4)	11.3 (10.8, 11.8)	5.9 (5.5, 6.2)	3.9 (3.7, 4)
32	Jewish AO	1573	253 (197, 315)	137 (112, 163)	19.2 (14.3, 25)	9.6 (7.6, 11.6)	0.4 (0.3, 0.5)	0.2 (0.2, 0.3)
33	Astrakhan	1351	252 (230, 274)	180 (169, 189)	22.9 (20.5, 25.4)	15.3 (14.3, 16.2)	2.5 (2.3, 2.8)	1.8 (1.7, 1.9)
34	Kirov	1670	248 (225, 269)	194 (185, 203)	17.4 (15.6, 19.2)	13.2 (12.5, 13.8)	3.1 (2.8, 3.4)	2.4 (2.3, 2.5)
35	Amur	1612	244 (218, 270)	245 (234, 256)	17.8 (15.7, 20.1)	17.9 (17, 18.9)	1.9 (1.7, 2.1)	1.9 (1.8, 2)
36	Tver	1842	243 (221, 267)	135 (124, 145)	15.2 (13.6, 16.9)	7.9 (7.2, 8.5)	3 (2.8, 3.3)	1.7 (1.6, 1.8)
37	Krasnodar	1428	242 (233, 252)	173 (169, 177)	20.4 (19.5, 21.4)	13.8 (13.5, 14.2)	13.7 (13.2, 14.3)	9.8 (9.6, 10)
38	Bryansk	1695	241 (218, 265)	163 (153, 174)	16.6 (14.8, 18.5)	10.7 (9.9, 11.4)	2.9 (2.6, 3.1)	1.9 (1.8, 2.1)
39	Pskov	1890	241 (208, 274)	137 (123, 151)	14.6 (12.4, 16.9)	7.8 (7, 8.7)	1.5 (1.3, 1.7)	0.9 (0.8, 0.9)
40	Khabarovsk	1535	237 (215, 257)	222 (214, 231)	18.2 (16.3, 20.1)	16.9 (16.2, 17.7)	3.1 (2.8, 3.4)	2.9 (2.8, 3)
41	Kostroma	1653	234 (203, 265)	132 (119, 146)	16.5 (14, 19.1)	8.7 (7.8, 9.7)	1.5 (1.3, 1.7)	0.8 (0.8, 0.9)
42	Karelia	1642	233 (201, 264)	168 (155, 181)	16.5 (14, 19.1)	11.4 (10.4, 12.4)	1.4 (1.2, 1.6)	1 (0.9, 1.1)
43	Kurgan	1724	232 (206, 259)	167 (155, 179)	15.5 (13.5, 17.7)	10.7 (9.8, 11.6)	1.9 (1.7, 2.1)	1.4 (1.3, 1.5)
44	Moscow	1173	231 (225, 237)	197 (195, 200)	24.6 (23.8, 25.4)	20.2 (19.9, 20.5)	29.3 (28.6, 30.1)	25 (24.7, 25.3)
45	Tomsk	1335	228 (207, 247)	201 (193, 210)	20.5 (18.3, 22.7)	17.7 (16.9, 18.7)	2.5 (2.2, 2.7)	2.2 (2.1, 2.3)
46	Smolensk	1706	226 (200, 253)	133 (121, 144)	15.3 (13.3, 17.4)	8.4 (7.6, 9.2)	2.1 (1.9, 2.4)	1.2 (1.1, 1.3)
47	Murmansk	1342	225 (199, 251)	207 (197, 218)	20.2 (17.4, 23)	18.2 (17.2, 19.4)	1.7 (1.5, 1.9)	1.5 (1.5, 1.6)
48	Belgorod	1543	225 (206, 243)	181 (173, 189)	17.1 (15.4, 18.7)	13.3 (12.6, 13.9)	3.5 (3.2, 3.7)	2.8 (2.7, 2.9)
49	Altai Krai	1630	223 (208, 240)	220 (214, 227)	15.9 (14.6, 17.2)	15.6 (15.1, 16.2)	5.2 (4.8, 5.5)	5.1 (4.9, 5.2)
50	Rostov	1532	222 (211, 233)	175 (170, 180)	16.9 (15.9, 17.9)	12.9 (12.5, 13.3)	9.3 (8.8, 9.8)	7.3 (7.1, 7.5)
51	Irkutsk	1490	204 (189, 219)	166 (160, 173)	15.8 (14.5, 17.3)	12.6 (12, 13.1)	4.9 (4.5, 5.2)	4 (3.8, 4.1)
52	Chechnya	620	203 (192, 215)	160 (155, 165)	48.9 (45, 53)	34.8 (33.3, 36.3)	3 (2.9, 3.2)	2.4 (2.3, 2.5)
53	Novgorod	1815	202 (167, 235)	111 (97, 124)	12.5 (10.1, 14.8)	6.5 (5.6, 7.4)	1.2 (1, 1.4)	0.7 (0.6, 0.7)
54	Krasnoyarsk	1409	194 (180, 207)	167 (161, 174)	15.9 (14.6, 17.2)	13.5 (12.9, 14)	5.5 (5.1, 5.9)	4.8 (4.6, 5)
55	Khakassia	1400	194 (161, 225)	125 (112, 138)	16 (13, 19.2)	9.8 (8.7, 10.9)	1 (0.9, 1.2)	0.7 (0.6, 0.7)
56	Kabardino-Balkaria	1004	193 (174, 214)	156 (147, 165)	23.8 (20.9, 27)	18.4 (17.1, 19.6)	1.7 (1.5, 1.9)	1.4 (1.3, 1.4)
57	Kemerovo	1610	192 (175, 207)	179 (173, 185)	13.5 (12.2, 14.7)	12.5 (12, 13)	5.1 (4.6, 5.5)	4.7 (4.6, 4.9)
58	Tyumen	1217	188 (170, 205)	113 (105, 121)	18.2 (16.2, 20.2)	10.2 (9.5, 11)	2.9 (2.6, 3.2)	1.7 (1.6, 1.9)
59	Karachay-Cherkessia	1063	186 (158, 217)	135 (122, 147)	21.3 (17.4, 25.6)	14.6 (13, 16.1)	0.9 (0.7, 1)	0.6 (0.6, 0.7)
60	North Ossetia	1199	185 (160, 209)	160 (149, 170)	18.3 (15.5, 21.1)	15.4 (14.2, 16.6)	1.3 (1.1, 1.5)	1.1 (1, 1.2)
61	Ivanovo	1763	181 (156, 206)	166 (155, 177)	11.4 (9.7, 13.3)	10.4 (9.6, 11.2)	1.8 (1.5, 2)	1.6 (1.5, 1.8)
62	Nenets AO	1033	180 (94, 268)	145 (106, 187)	21 (10, 35.1)	16.3 (11.4, 22.1)	0.1 (0, 0.1)	0.1 (0, 0.1)
63	Dagestan	641	180 (172, 188)	135 (131, 138)	38.9 (36.7, 41.4)	26.7 (25.8, 27.5)	5.6 (5.4, 5.9)	4.2 (4.1, 4.3)
64	Primorsky Krai	1527	179 (162, 197)	180 (173, 187)	13.3 (11.9, 14.8)	13.3 (12.8, 14)	3.4 (3.1, 3.7)	3.4 (3.3, 3.5)
65	Arkhangelsk	1476	179 (157, 202)	137 (127, 147)	13.8 (11.9, 15.8)	10.3 (9.4, 11)	1.9 (1.7, 2.2)	1.5 (1.4, 1.6)
66	Stavropol	1297	177 (164, 189)	151 (145, 157)	15.8 (14.4, 17.1)	13.2 (12.6, 13.7)	4.9 (4.6, 5.3)	4.2 (4.1, 4.4)
67	Kamchatka	1263	176 (136, 214)	137 (121, 154)	16.2 (12.1, 20.4)	12.2 (10.6, 13.9)	0.6 (0.4, 0.7)	0.4 (0.4, 0.5)
68	Komi	1339	169 (144, 193)	132 (121, 142)	14.5 (12.1, 16.8)	10.9 (10, 11.8)	1.4 (1.2, 1.6)	1.1 (1, 1.2)
69	Sakhalin	1376	163 (129, 196)	104 (89, 118)	13.5 (10.4, 16.6)	8.2 (6.9, 9.4)	0.8 (0.6, 1)	0.5 (0.4, 0.6)
70	Kalmykia	1106	161 (124, 198)	134 (116, 150)	17.1 (12.6, 21.8)	13.7 (11.8, 15.7)	0.4 (0.3, 0.5)	0.4 (0.3, 0.4)
71	Sakha (Yakutia)	918	161 (143, 179)	104 (96, 112)	21.3 (18.4, 24.2)	12.8 (11.7, 13.9)	1.6 (1.4, 1.7)	1 (0.9, 1.1)
72	Khanty-Mansiysky AO	760	157 (145, 170)	138 (133, 143)	26.2 (23.6, 28.8)	22.1 (21.2, 23.2)	2.6 (2.4, 2.9)	2.3 (2.2, 2.4)
73	Altai Republic	1129	157 (112, 201)	114 (93, 133)	16.1 (11, 21.7)	11.2 (9, 13.4)	0.3 (0.2, 0.4)	0.3 (0.2, 0.3)
74	Yamalo-Nenets AO	602	155 (136, 174)	106 (97, 114)	34.8 (29.2, 40.5)	21.3 (19.2, 23.5)	0.8 (0.7, 0.9)	0.6 (0.5, 0.6)
75	Kaliningrad	1310	154 (133, 178)	64 (54, 74)	13.4 (11.3, 15.7)	5.1 (4.3, 6)	1.6 (1.3, 1.8)	0.6 (0.5, 0.7)
76	Vologda	1543	154 (131, 177)	89 (79, 98)	11.1 (9.3, 13)	6.1 (5.4, 6.8)	1.8 (1.5, 2)	1 (0.9, 1.1)
77	Zabaykalsky Krai	1349	150 (128, 171)	117 (108, 127)	12.5 (10.5, 14.5)	9.5 (8.7, 10.4)	1.6 (1.4, 1.8)	1.2 (1.1, 1.3)
78	Tuva	914	146 (111, 180)	-6 (-21, 9)	19 (13.8, 24.5)	-0.7 (-2.2, 1)	0.5 (0.4, 0.6)	0 (-0.1, 0)
79	Magadan	1258	124 (66, 180)	106 (82, 129)	10.9 (5.5, 16.7)	9.2 (6.9, 11.5)	0.2 (0.1, 0.3)	0.1 (0.1, 0.2)
80	Buryatia	1174	109 (88, 131)	73 (64, 82)	10.3 (8.1, 12.6)	6.7 (5.8, 7.5)	1.1 (0.9, 1.3)	0.7 (0.6, 0.8)
81	Adygea	1313	106 (71, 141)	49 (35, 64)	8.8 (5.7, 12)	3.9 (2.7, 5.1)	0.5 (0.3, 0.7)	0.2 (0.2, 0.3)
82	Ingushetia	387	87 (71, 104)	67 (60, 74)	29.1 (22.4, 36.6)	20.9 (18.3, 23.8)	0.4 (0.4, 0.5)	0.3 (0.3, 0.4)
83	Chukotka	1033	45 (-48, 137)	52 (14, 91)	4.5 (-4.4, 15.3)	5.3 (1.3, 9.6)	0 (0, 0.1)	0 (0, 0)

Notes: Method A – averages in the same months in preceding years; Method B – averages adjusted for secular trends.

* Regions are listed in descending order by excess death rates (method B).

## Footnotes

1 A new comparative study was published at the time this paper has been accepted for publication (Islam et al., 2021). It shows that the highest reduction in life expectancy among 37 countries was observed in Russia in 2020 (−2.33 and −2.14 years for males and females, respectively).

2 The list of comparator countries contains those countries that are included in Human Mortality Database and thus meet the requirements of having high quality statistical systems. The exact list of countries can be found in Table A1 in Appendix.

3 STMF is a new open-access resource that was launched in May 2020 by the Human Mortality Database team in the response to the COVID-19 pandemic, and provides comparable and harmonized weekly death counts (Human Mortality Database, 2021; Jdanov et al., 2021).

4 Based on the assumption that deaths are equally distributed within a week.

5 We applied the Lee-Carter method to forecast population-at-risk exposure for the most recent country-years that were not available in the HMD (Lee & Carter, 1992).

6 These data are included in the statistical form No. 12 “Information on the number of diseases registered in patients living in the service area of a medical organization”, and were obtained from Federal Research Institute for Health Organization and Informatics of Ministry of Health of the Russian Federation upon request. Accessed September 27, 2021.

7 The observed number of deaths in each month was multiplied by the length of the standard month, i.e 30.4375 days, and divided by the length of each month in days. A value of 28.25 days was used for February.

8 We don't include January and February in our analysis as there were almost no excess deaths due to COVID-19 over this period, but enlarge the second quarter by adding March.

9 Our additional calculations from publicly available data (National Center for Health Statistics data for the USA; Eurostat, INED and data.gouv.fr data for France) also find a positive correlations between excess mortality and the cumulative number of COVID-19 cases in 2020 across 50 states and the District of Columbia of the USA (r = 0.44, p < 0.001) and 13 regions of France (r = 0.82, p < 0.001).
